# Structural Integrity and Functional Neural Activity Associated with Oral Language Function after Stroke

**DOI:** 10.3390/jcm11113028

**Published:** 2022-05-27

**Authors:** Sunghyon Kyeong, Hyunkoo Kang, Dae Hyun Kim

**Affiliations:** 1 Institute of Behavioral Science in Medicine, Yonsei University College of Medicine, Seoul 03722, Korea; sunghyon.kyeong@gmail.com; 2 Department of Radiology, Veterans Health Service Medical Center, Seoul 05368, Korea; knroo@bohun.or.kr; 3 Department and Research Institute of Rehabilitation Medicine, Yonsei University College of Medicine, Seoul 03722, Korea

**Keywords:** stroke, recovery, aphasia, connectivity

## Abstract

(1) Background: The impairment of language function after a stroke is common. It is unclear how the brain reorganizes for language function after cerebral infarction. The aim of this observational study is to investigate the association of structural integrity and functional neural activity with language function in aphasic patients with middle cerebral artery infarction. (2) Methods: Magnetic resonance images and scores from the Western Aphasia Battery on 20 patients were retrieved from medical records. A Voxel-wise linear regression analysis was performed using fractional anisotropy maps or the fractional amplitude of low-frequency fluctuation maps as dependent variables and scores of oral language function as independent variables while controlling for age and time elapsed after stroke. (3) Results: Spontaneous speech was positively associated with fractional anisotropy in the left dorsal stream and the right posterior corpus callosum and with the fractional amplitude of the low-frequency fluctuation of cranial nuclei in the pontomedullary junction. Comprehension was positively associated with the left ventral stream. Naming was positively associated with the left ventral stream and the bilateral occipitofrontal fasciculus, as well as with the fractional amplitude of low-frequency fluctuation of the supramarginal gyrus in the left hemisphere. (4) Conclusions: The dorsal and ventral streams are important for articulation and meaning after the reorganization of neural circuits following stroke. Subdomains of oral language function with a visual component are dependent on the visual association areas located in the right hemisphere.

## 1. Introduction

Aphasia is a common impairment after left hemispheric stroke. Stroke survivors with aphasia experience problems communicating with other individuals and participating in social activities [1,2]. The prevalence of aphasia after stroke ranges from 25% to 50% [3]. Discrepancies in the reported prevalence are due to the use of different definitions or evaluation methods. Aphasia represents a multimodal language dysfunction that cannot be simply identified by clinical screening.

Language function associated with large-scale networks is organized in specialized cortical areas of the brain and their interconnecting white matter fiber tracts. With the advent of functional magnetic resonance imaging (fMRI) and diffusion tensor imaging (DTI), the classic language model based on Broca’s and Wernicke’s areas, and the white matter fibers connecting these two brain regions, has been challenged [4,5]. In its place, a novel dual-stream language model has been proposed to study language processing, in which a dorsal pathway is concerned with the transition from sound to articulation and a ventral pathway with that from sound to meaning [4,6,7,8,9]. This model pays more attention to the connection between the cortical areas and explains the effect of cortical injury and disconnection on functions typically compromised in aphasia, such as fluency, naming, comprehension, and repetition [10]. However, the neuroanatomical models of language organization constitute limitations for the treatment of aphasia after stroke. The dynamic reorganization that takes place after stroke affects the course of recovery in aphasic patients [11,12].

The neuronal reorganization that takes place after brain injury contributes to the recovery of language function. Understanding neuronal reorganization associated with recovery from aphasia would, therefore, help define goals and the appropriate treatment after stroke. A previous study using serial fMRI illustrates the reorganization process of language function that takes place after a stroke episode [11]. This process includes an initial decrease of cortical activity in the language areas of the left hemisphere in parallel with increased activity of the homologous areas in the right hemisphere and a re-shift of peak activation to the left hemisphere at later stages of recovery [11]. This implies that uninjured language networks in the left hemisphere primarily contribute to recovery, and other study results support this proposed mechanism [13,14,15,16]. However, there are several limitations in previous studies focused on the reorganization of neural networks associated with the recovery of language function. First, large left hemispheric lesions, which may require the recruitment of the right hemisphere, or cases of incomplete recovery were not included [6,12,17,18]. Second, the dynamic network changes were studied almost exclusively using fMRI, with only a few studies having taken advantage of DTI to investigate the reorganization in structural integrity associated with aphasia recovery [6,19,20]. Third, aphasia represents a multimodal impairment of language function, which includes deficits in fluency, comprehension, naming, and repetition. Previous studies have focused on specific subdomains of language function, but each subdomain may depend on different structural and functional changes and reorganization processes in the path to recovery of language function.

This study aimed to investigate the brain areas associated with each subdomain of language function in patients with chronic mild-to-severe aphasia resulting from middle cerebral artery infarction. The DTI and resting-state fMRI (rsfMRI) techniques were used to study the associated brain regions in terms of structural integrity and functional neural activity, respectively.

## 2. Materials and Methods

### 2.1. Patients

**Figure 1:**
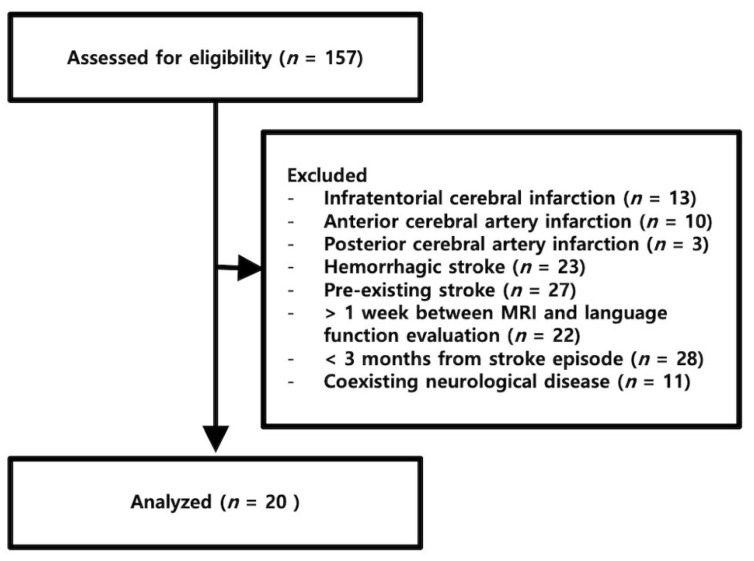
Flowchart of the study process. Flow diagram depicting the reasons for exclusion among the patients assessed for eligibility.

We retrospectively reviewed the medical records of 157 patients admitted to the Veterans Health Service Medical Center in Seoul (Republic of Korea) for inpatient rehabilitation and who underwent DTI and rsfMRI between May 2015 and May 2020. The inclusion criteria were (a) first ever stroke, as confirmed by MRI; (b) native Korean speaker; (c) left middle cerebral artery infarction; (d) right-handedness before stroke; (e) more than three months elapsed since the episode of cerebral infarction; and (f) MRI and language evaluation having been performed within a 1-week interval, and the order was not limited. The exclusion criterion was any co-existing neurological disease capable of influencing language function. Twenty patients met the inclusion criteria and were, therefore, enrolled in this retrospective study (Figure 1). All patients were men and received inpatient rehabilitation, including speech therapy. 

### 2.2. Language Evaluation

Language evaluation results obtained using the Korean version of the Western Aphasia Battery (WAB) were retrieved from the medical records [21]. The WAB is a standardized tool used for testing aphasia in English-speaking countries [21]. The WAB consists of subtests evaluating four oral language areas, two written language areas, and other cognitive abilities [21]. The translated Korean version of WAB (K-WAB) has been validated and used for the Korean population [21]. We used scores corresponding to four different oral language areas: spontaneous speech, comprehension, repetition, and naming. In the K-WAB, spontaneous speech is assessed both through responses to questioning and based on the patient’s description of a line-drawing picture. Comprehension is assessed based on closed questioning (yes/no responses), auditory word recognition, and responses to sequential commands. Repetition is assessed based on the individual’s ability to repeat the names of a variety of items, and naming is assessed based on object naming, sentence completion, word fluency, and responsive speech. The reference values for the K-WAB have been reported previously [21]. Lower scores indicate poorer language function.

### 2.3. MRI Preprocessing

All MRI scans were acquired using a 3-T MR scanner (Siemens, Erlangen, Germany) with a 20-channel head coil. High-resolution 3D T1-weighted images, DTI, and rsfMRI scans were obtained. Detailed parameters for the MRI scans are shown in Table 1. For lesion volume measurements, a neuroradiologist drew each patient’s lesion on T1-weighted images in the native space using the 3D slicer software [22]. For further analysis, DTI was registered to the corresponding b = 0 image using an affine transformation to correct distortions caused by eddy currents (FSL 5.0.10; http://www.fmrib.ox.ac.uk/fsl, accessed on 1 August 2017, FMRIB Analysis Group, Oxford, UK). Then, fractional anisotropy (FA) maps were constructed in native space and co-registered to the individual’s T1-weighted image. For the voxel-wise analysis of FA maps, T1-weighted MR images and the co-registered FA images were nonlinearly normalized to the standard Montreal Neurological Institute (MNI) space.

The rsfMRI underwent motion correction by realignment with the middle volume of each run, spatial normalization to the MNI space, and smoothing with a Gaussian kernel of 8 mm full-width at half maximum. Subsequently, the rsfMRI scans were temporally detrended. To remove confounding effects, we regressed out artifacts from head motions and physiological noises from the cerebrospinal fluid and white matter. After regressing out all nuisance parameters, residual time-series data were transformed to the frequency domain for evaluating the amplitude of low-frequency fluctuation (ALFF). Finally, the voxel-wise fractional ALFF (fALFF) was measured as a ratio of the power spectrum at the low-frequency range (0.009–0.08 Hz) to that of the entire frequency range [23].

### 2.4. Statistical Analysis

A Voxel-wise linear regression analysis for the white matter and the gray matter areas was performed using transformed FA and fALFF maps as dependent variables and language scores (spontaneous speech, comprehension, repetition, or naming) as independent variables, while controlling for age and duration after stroke. Significant associations between maps and language scores were identified by applying cluster-level family-wise-error corrected *p* < 0.05 with a cluster-forming threshold of *p* < 0.001 at each voxel [24]. In a post-hoc analysis, we extracted the regional mean values for the brain areas significantly associated with language scores. We subsequently illustrated the relationships and computed the correlation values between the regional neuroimaging values and language scores.

## 3. Results

### 3.1. Patient Characteristics

**Figure 2:**
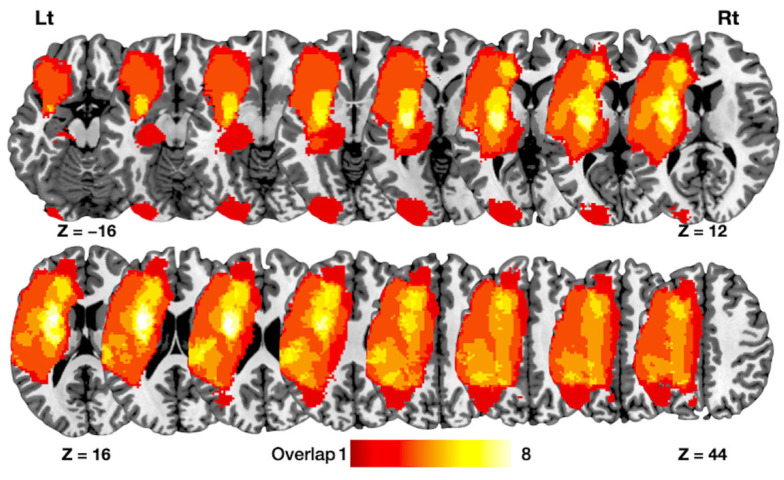
Lesion overlapping maps. Overlay map showing the lesion location in each of the patients 20 included in the study. A T1-weighted map was used to demarcate the lesion corresponding to each patient. The color scale indicates the degree of lesion overlap between patients, with the maximum overlay corresponding to eight patients. Rt = right hemisphere. Lt = left hemisphere. z = *z*-axis in Montreal Neurological Institute space.

The average age (mean ± standard error) of the patients was 71.9 ± 2.4 years, and the average duration between cerebral infarction and MRI evaluation was 1219.7 ± 432.6 days. The average language scores of the patients were as follows: Spontaneous speech was 11.1 ± 1.7, comprehension was 6.5 ± 0.7, repetition was 6.0 ± 1.0, and naming was 5.5 ± 0.9 (Table 2). The average lesion volume was 51,249.2 ± 19,790.8 mm^3^. A lesion overlay map for all included patients is shown on Figure 2.

### 3.2. Structural Atrophy Associated with Language Function

**Figure 3:**
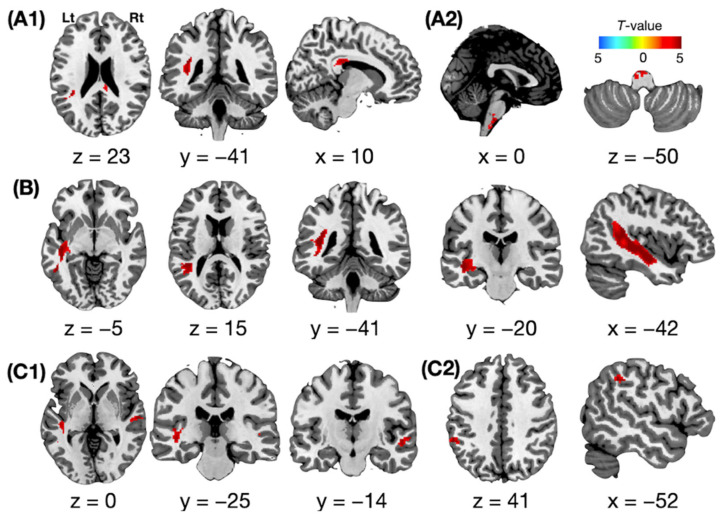
Representative images of brain maps associated with specific subdomains of language function. Voxel-wise linear regression analyses revealed that brain maps based on fractional anisotropy (FA) and the fractional amplitude of low-frequency fluctuation (fALFF) show a significant association with four different oral language areas: A positive association between FA and spontaneous speech (**A1**); a positive association between fALFF and spontaneous speech (**A2**); a positive association between FA and comprehension (**B**); a positive association between FA and naming (**C1**); and a positive association between fALFF and naming (**C2**). Statistical threshold: cluster-level family-wise-error corrected *p* < 0.05. The red scale indicates positive association, and the blue scale indicates negative association. Rt = right hemisphere. Lt = left hemisphere. x, y = x-, y-axis in Montreal Neurological Institute space.

**Figure 4:**
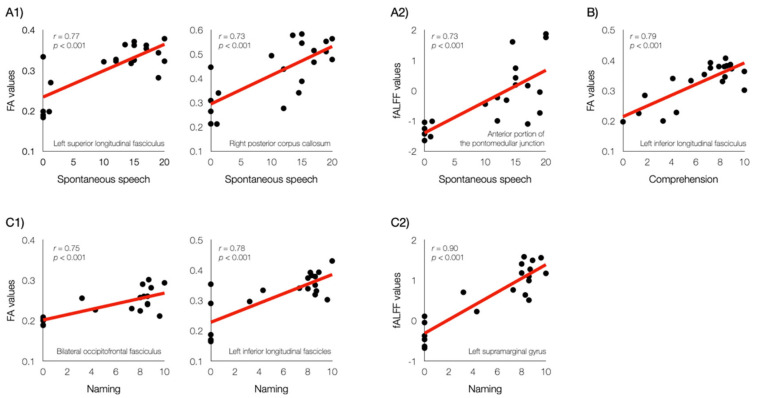
Relationships between regional neuroimaging values and language scores. (**A1**), positive association between regional fractional anisotropy (FA) values and spontaneous speech; (**A2**), positive association between regional fALFF values and spontaneous speech; (**B**), positive association between regional FA values and comprehension; (**C1**), positive association between regional FA values and naming; (**C2**), positive association between regional fALFF values and naming.

Spontaneous speech was positively associated with the FA values from the left superior longitudinal fasciculus and the right posterior corpus callosum. Comprehension was positively associated with the FA values of the left inferior longitudinal fasciculus. Naming was positively associated with the FA values of the left inferior longitudinal and bilateral occipitofrontal fasciculus (corrected *p* < 0.05, Figure 3 and Figure 4). No brain regions were positively associated with repetition, and no negative association with language function was observed for any brain region.

### 3.3. Functional Neural Activity Associated with Language Function

Spontaneous speech was positively associated with the fALFF values of the anterior portion of the pontomedullary junction. Naming was positively associated with the fALFF values of the supramarginal gyrus in the left hemisphere (corrected *p* < 0.05, Figure 3 and Figure 4). No brain region was positively associated with comprehension or repetition, and no negative association with language function was observed for any brain region.

## 4. Discussion

We reveal in this study some specific brain areas positively associated with performance in four different subdomains of oral language function after a middle cerebral artery infarction. The association between language function and brain regions was analyzed after the reorganization process had presumably reached a plateau, since only patients who were tested three or more months after stroke onset were included in the study. Dorsal and ventral pathways in the left hemisphere were positively associated with spontaneous speech and comprehension, respectively, whereas naming was positively associated with the ventral pathway and with the occipitofrontal fasciculus in the left hemisphere. Furthermore, the posterior corpus callosum and the occipitofrontal fasciculus in the right hemisphere were positively associated with spontaneous speech and naming, respectively. The grey matter activity in the brainstem and the supramarginal gyrus was positively associated with spontaneous speech and naming, respectively.

The DTI technique measures the directional diffusion of water along bundles of myelinated axons. FA values indicate increased water diffusion along axons, which, in turn, reflects enhanced structural integrity of subcortical regions [6,25]. On the other hand, the rsfMRI signal is based on the spontaneous fluctuations of oxygen levels in the blood. Most studies based on rsfMRI measurements demonstrate the functional connectivity of spatially distinct brain areas using this signal [26,27,28]. However, it should be kept in mind that functional connectivity does not provide direct information on brain activity, only on the functional correlation between different brain regions [23]. As an alternative, we used fALFF values, which are indicators of functional neural activity in the grey matter region. The regression analysis using brain maps derived from FA and fALFF values, thus, reflects the structural integrity in the white matter and the functional neural activity in the grey matter associated with language function.

The structural integrity of the dorsal and ventral pathways plays important roles in the reorganization process after stroke according to the dual-stream language model. The dorsal pathway, which includes the superior longitudinal fasciculus, is involved in auditory-motor integration by matching sounds from acoustic speech to articulatory representation [4,7]. On the other hand, the ventral pathway, represented by the inferior longitudinal fasciculus, is where sound and meaning are integrated. The subdomains of spontaneous speech and comprehension roughly reflect the functions of articulation and matching between sound and meaning, respectively. The roles for both pathways suggested by our results are in line with those of previous studies [4,7], and the association with language functions is still significant after reorganization triggered by stroke in aphasic patients.

The specific role of the right hemisphere is dependent on the language subdomain being considered. The spontaneous speech and naming subdomains were demonstrated, in this study, to have a significant association with brain regions in the right hemisphere, including the posterior corpus callosum and the occipitofrontal fasciculus. The evaluation processes for these two subdomains commonly include visual inputs, such as in picture descriptions and object naming. The brain regions in the right hemisphere that showed positive associations are related to the visual pathway. The regions of the posterior corpus callosum that exhibited a positive association in this study contain fibers passing from the temporal to the occipital lobes [29]. The occipitofrontal fasciculus connects the occipital, temporal, and frontal lobes. Visual inputs are recognized by the occipital lobes in both hemispheres and interface with conceptual systems in the ventral pathway for object recognition. The language function is lateralized to the left hemisphere, and the activation of the right hemisphere during the process of recovery from stroke is indicative of decreased efficiency [11,13,16]. However, the visual system distributes bihemispheric networks and may contribute to oral language function [30,31]. The role of the right hemisphere in language recovery after stroke may depend on these bihemispheric networks that includes the visual system.

The association observed between neural activity and the subdomains of oral communication reflects the roles of specific brain regions in language function. Cranial nerves V, VII, IX, X, XI, and XIl play roles in speech production [32]. An articulatory motor system finally projects to the cranial nuclei in the brainstem, which innervates the bulbar muscles required for phonation via the corticobulbar tracts. The association between neural activity at the pontomedullary junction and spontaneous speech reflects the integration of auditory–motor processes required for articulation. Furthermore, perilesional activity in the left hemisphere enhances the process of recovery after stroke [12,33,34]. The process of naming starts on the visual system and passes to the angular gyrus at the junction of the occipital, parietal, and temporal lobes. Then, the context provided in the angular gyrus transforms the signal into an evoked output for the motor cortex. In order to achieve the recovery of language function, it appears that other cortical areas (the supramarginal gyrus in this case) often compensate for what is lost.

There were no brain regions showing a negative association with language function in the present study. A possible explanation may be due to the inclusion criteria used. We only included chronic patients, with an average lapse of 1219 days after stroke. Dynamic changes, such as diaschisis, compensation, cortical reorganization, and subcortical reconnection, occur in both hemispheres immediately after stroke [12,33]. Negative associations are more likely to be evident in the acute-to-subacute phases after stroke. However, it should be mentioned that the sample size was relatively small, and the duration between the stroke episode and the testing was heterogeneous in the present study. A prospective study focused on structural and functional aspects of language recovery after stroke is warranted.

## 5. Conclusions

The dual pathway supporting language function is still important for spontaneous speech and comprehension after the reorganization that follows a stroke episode. The functions of oral language that involve visual processing are influenced by the visual association areas located in the right hemisphere. Activity patterns in the grey matter reflect neural pathways associated with specific subdomains of oral language function.

## Figures and Tables

**Table 1:** Magnetic resonance imaging (MRI) data acquisition.

Parameter	T1-Weighed MRI	DTI	rsfMRI
Matrix	256 × 256	112 × 112	64 × 64
Field of view (mm^2^)	230 × 230	224 × 224	192 × 192
Repetition time (ms)	1900	9700	2000
Echo time (ms)	2.57	92.00	30.00
Slice thickness (mm)	1	2	4.4
Flip angle (°)	9	9	9
Directions		30	
*b* values (s/mm^2^)		1000	

DTI, diffusion tensor imaging; rsfMRI, resting state functional magnetic resonance imaging.

**Table 2:** Demographic characteristics and language function scores of patients included in the study.

	(*n* = 20)/Mean ± SE/*n* (%)
Demographic characteristics	
Age (years)	71.9 ± 2.4
Duration between stroke and performed MRI (days)	1219.7 ± 432.6
Language evaluation (K-WAB)	
Spontaneous speech (20)	11.1 ± 1.7
Comprehension (10)	6.5 ± 0.7
Repetition (10)	6.0 ± 1.0
Naming (10)	5.5 ± 0.9
Aphasia severity	
Mild	6 (30.0%)
Mild to moderate	6 (30.0%)
Moderate	1 (5.0%)
Moderate to severe	0 (0%)
Severe	7 (35.0%)

K-WAB, Western Aphasia Battery (Korean version), SE, standard error.

## Data Availability

The data presented in this study are available on request from the corresponding author. The data are not publicly available due to privacy.
